# 

**Published:** 1894-06

**Authors:** 


					﻿THE
Southern Medical Record.
A Monthly Journal of Medicine and Surgery.
Vol. XXIV.
ATLANTA, GA., JUNE, 1894.
No. 6.
EDITORS ■
A. W. GRIGGS, M. D.
j. McFadden gaston, m. d.
WILLIS F. WESTMORELAND, M. D,
LOUIS H. JONES, M. D.
DAN. H. HOWELL, M. D., Business Manager.
TERMS: $2.00 Per Annum ; Single Copy 20 Cents.
Contents on Page 5
Entered at the Postoffice at Atlanta, Ga., as Second-Class Matter.
Tne Franklin Printing and Publishing Co., Atlanta, Ga.
				

